# Supplementary material for: Are carbon emissions trading and green financial instruments synergistic? -Comprehensive quantitative research based on content analysis
Source: PLoS One. 2024 Mar 7;19(3):e0298601. doi: 10.1371/journal.pone.0298601 (PMC10919601; doi:10.1371/journal.pone.0298601)
Supplement: S1 Data — (ZIP) [file pone.0298601.s001.zip › Data/地级市Z_F工作报告.zip!/╦─┤¿/└╓╔╜╩╨/└┤╫╘╣1⁄2╓┌║┼í╛┬φ┐╦╩2╛▌═°í┐_2014.docx]

各位代表：

　　现在，我代表乐山市人民政府，向大会报告工作，请予审查，并请市政协各位委员提出意见。

　　2013年工作回顾

　　刚刚过去的2013年，是充满挑战、攻坚克难的不平凡之年。在市委的坚强领导下，在市人大、市政协的监督支持下，我们牢固树立科学发展、加快发展的工作指导思想，全面实施“三大发展战略”，务实推进“三大联动、六个跨越、六个美丽”，紧紧围绕“改革开放年”主题，积极有效应对各种复杂形势和多重灾害挑战，各项工作取得了新的成绩。全市实现地区生产总值1134.8亿元、增长10.4%；地方公共财政收入75.1亿元、增长6.7%；城镇居民人均可支配收入22661元、增长11.1%，农民人均纯收入8737元、增长12.8%。

　　一、稳定增势有力有效

　　一年来，我们沉着应对经济下行压力，保持定力抓发展，精准发力稳增长，及时制定实施稳投资、抓工业、促消费等政策措施，促进了经济平稳向好发展。

　　投资拉动更加有力。狠抓重大项目建设，完成全社会固定资产投资787亿元、增长20.8%。产业化项目投资力度加大，科创生物医药产业园、农夫山泉饮料生产基地等项目开工建设，和邦15万吨双甘膦、玖龙30万吨高强瓦楞纸等项目竣工投产。全域交通建设强力推进，乐雅、乐自高速、国道213线犍为和沐川绕城段、省道103线和306线峨眉过境段、省道305线乐夹路改造等项目建成通车。能源水利建设得到加强，毛滩水电站并网发电，仁寿—乐山天然气管线建成，安谷、枕头坝一级、沙坪二级等水电站和金王寺水库枢纽加快建设。

　　工业经济企稳向好。采取减轻企业负担、加强市场开拓、保障生产要素等综合措施，“一企一策”强化企业帮扶，工业企业产能发挥率由低谷时的75%提高到90%左右，产销率由95.9%回升到97.3%。工业“双千双百”培育工程启动实施，沙湾不锈钢产业园区成为国家新型工业化产业示范基地，五通桥盐磷精细化工循环产业园区成为全省第二批新型工业化产业示范基地，夹江经济开发区完成扩区调位，井研、犍为工业集中区跻身“百亿园区”，福华集团成为“百亿企业”，主营业务收入超50亿元的企业集团新增1家、超20亿元的新增2家。全市工业实现增加值649.8亿元、增长11.2%。

　　文旅消费繁荣活跃。峨眉成为全国首批旅游综合改革试点县（市），郭沫若故居创建为4A级旅游景区，成功申报省级水利风景区5个。第五届乐山大佛旅游文化节、第四届峨眉武术节等活动成功举办，累计接待国内外游客2994.9万人次，实现旅游综合收入319.8亿元，分别增长12%和19%。大力繁荣城乡消费，举办“乐山造”品牌商品展销等会展促销活动，全市限额以上批发零售和住宿餐饮单位达474家、净增153家，实现社会消费品零售总额389.6亿元、增长14.5%。信息消费持续升温，智能手机、宽带接入、数字电视等终端用户新增11.2万户，成功申报为国家首批信息消费试点市。

　　二、发展质量得到提高

　　一年来，我们坚持以发展质量和效益为中心，不失时机转方式、调结构，实现了稳中有进、稳中提质。

　　产业升级步伐加快。新兴产业加速壮大，占全市规模以上工业企业主营业务收入的比重达31%、提高3个百分点。国家硅产品质量监督检验中心暨乐山高新区公共检测服务平台建成投用。传统产业改造升级，德胜集团、西南不锈钢公司和永祥多晶硅公司成为全国行业规范条件达标企业，淘汰落后产能重点项目18个。煤矿企业清理整顿和兼并重组工作走在全省前列，完成45处小煤矿关闭。全市创中国驰名商标14件、新增3件。确立建设四川金融次中心目标，市商业银行自贡、资阳、南充分行开业，浙商银行、平安银行到乐山开设分支机构，全市银行业金融机构本外币各项存款余额1526.7亿元，贷款余额934.1亿元，分别新增261.9亿元和124.8亿元。现代农业加快发展，建成现代农业万亩示范区24个，农业产业化经营带动农户面提高到65%。沐川富和乡跻身全国“一村一品”示范村镇，“犍为茉莉花茶”成功注册国家地理标志集体商标，新创国家级农民示范专业合作社、国家地理标志保护产品各3个。全市粮食总产量实现七连增。

　　科技创新能力增强。国家高新技术企业达38家、新增6家，省级工程技术研究中心达12个、新增4个。高新技术产业实现产值238.6亿元、增长45%。新获专利授权542件，其中发明专利授权80件。盛和稀土公司“少铈氯化稀土、氟化铈一步生产法”发明专利获得中国专利优秀奖。乐山国家农业科技园区第二轮规划启动实施，获科技部评定为优良园区。全市连续5届评为全国科技进步先进市，五通桥、犍为评为2013年全国科技进步先进县（区）。

　　新型城镇加快建设。中心城区新区开发有序推进，青江新区至乐路、临港新城“两横”道路完成主体工程，岷江东岸景观大道竣工通车，嘉州长卷滨江湿地景观建成开放。老城功能得到完善，海棠路改建、绿海路新建及白岩竹溪文化公园一期等项目竣工投用，岷江二桥拓宽维修工程基本完工，嘉兴路等4条断节路贯通，长江市场等3处人行天桥建成通行。城市基础设施日臻完善，品位进一步提升。各县（市、区）新区开发、老城综合整治深入实施，11个省、市试点示范小城镇加快建设，12个新农村综合体示范试点项目基本建成。全市新增城镇建成区面积8.4平方公里，城镇化率达44.53%、提高1.56个百分点。

　　生态建设力度加大。加强生态保护与建设，全市新增森林蓄积量107万立方米，森林覆盖率提高到54.25%。沐川、峨边、马边纳入全省重点生态功能区，市中区创建为省级生态区，15个乡镇创建为省级生态乡镇。节能减排有力推进，完成减排项目89个，建成乡镇生活污水处理设施29处。全年单位地区生产总值能耗下降5.01%，能够完成省下达的节能减排目标。城乡环境综合治理深入实施，峨眉成为全省环境优美示范县城，沙湾嘉农镇等6个乡镇、马边烟峰村等50个村分别创建为全省环境优美示范乡镇和示范村庄。

　　三、改革开放持续深化

　　一年来，我们紧紧围绕“改革开放年”主题，坚定不移深化改革、扩大开放，增强了发展动力活力。

　　各项改革加快推进。行政审批制度改革得到深化，市级行政审批项目精简182项、非行政许可审批项目精简39项，保留的审批事项实际办理提速88.2%，行政权力依法规范公开运行平台开通运行。农村土地承包经营权确权登记颁证全面启动，农村土地流转面积达30万亩，农业适度规模经营比重提高到16.8%。户籍管理制度改革启动实施，全市城镇落户实现“零门槛”，成为省内首个发放居住证的二级城市。市、县食品药品监管体制改革完善任务基本完成。事业单位分类改革有序推进。“营改增”试点、基本医保付费方式改革等顺利实施，县级公立医院实行药品“零差价”销售。市公共资源交易服务中心挂牌成立。

　　开放招商成果丰硕。借助民企入川、央企入川、港澳企业入川、华商大会、西博会等平台，深入实施“千亿招商”工程，新签约正式合同项目184个、投资总额1637.8亿元。万达集团、王府井百货等国内外知名企业成功引进，全市引进到位市外内资560亿元，其中省外内资406亿元，分别增长21.7%和31%。对外贸易迈上新台阶，创建省级外贸发展示范基地3个，外贸进出口总额11.2亿美元，其中出口8.3亿美元，分别增长18.4%和25.5%。

　　民营经济健康发展。改革工商登记制度，放宽市场主体注册登记及出资条件，深入实施中小企业成长工程和中小微初创企业重点扶持工程，积极鼓励和支持民营经济又好又快发展。全市非公经济主体（含个体户）达11.5万个、新增7384个，其中私营企业13784家、新增1822家。三八商场连锁等5家企业获全省“优秀民营企业”称号。

　　四、群众生活不断改善

　　一年来，我们坚持把人民利益放在首要位置，持续加大民生投入，不断加强社会建设，维护了社会和谐稳定。

　　抗灾重建科学高效。面对突如其来的芦山强烈地震，我们坚持“生命至上”，奋力抗灾自救，紧急驰援灾区，严防次生灾害，妥善安置受灾群众6169人，帮助芦山重灾区搜救转移群众7748人、救治伤病员2307人。面对暴雨洪涝灾害，我们全体动员，有针对性地提前落实防汛减灾措施，临时转移安置群众8.7万人，劝返疏散游客1.5万人，实现了“零死亡、零群伤”目标。科学编制实施灾后恢复重建规划，全面完成12940套城乡住房维修加固，完成城乡住房重建766套。

　　民生工程深入实施。“十项民生工程”项目和新增民生事项年度任务全面完成，投入民生工程资金54.6亿元，全市民生支出占公共财政支出的比重达65.4%。狠抓就业创业工作，城镇新增就业4.2万人，城镇登记失业率为3.8%。城乡居民大病保险、重特大疾病医疗救助、高龄补贴制度普遍建立。企业退休人员养老金、最低工资标准、工伤保险待遇、农村五保供养标准进一步提高。大力推进扶贫攻坚，在180个村实施彝家新寨、整村推进等专项扶贫项目，5.9万群众实现脱贫。大中型水利水电工程移民工作有序推进，实施生产安置2932人、搬迁安置2222人。开工建设保障性安居工程7712套、竣工12748套，农村危房改造完成9599户。保障性安居工程项目“斑竹印象”荣获中国土木工程詹天佑奖。解决了18万农村人口饮水安全问题。对口援藏扎实开展，理塘县城群众应急避难场所、甲洼乡特色紫皮马铃薯示范种植基地建成投用，理塘县城自来水工程项目开工建设。

　　社会事业全面发展。教育事业优先发展，学前教育三年行动计划圆满完成，博睿特外国语学校建成投用，乐山一职中创建为国家首批中职教育改革发展示范学校，乐山职业技术学院成为省级示范性高等职业院校立项建设院校。教育助学政策全面落实，民族地区义务教育阶段学生营养改善计划实现全覆盖。卫生工作得到加强，新创建省级数字化医院3家、三乙医疗保健机构4家、二甲中医医院5家，全市实现血吸虫病传播阻断目标，井研创建为省级卫生县城，首期民族医士班开班。人口自然增长率为2.26‰。文体事业协调发展，成功举办市第六届运动会和首届残疾人田径运动会，“文瀚嘉州·百姓直通车”列为国家公共文化服务体系示范项目，井研三江白塔、市中区离堆跻身全国重点文物保护单位，犍为县图书馆成为全省首批古籍重点保护单位。第三次经济普查工作顺利开展。31个乡镇补建了邮政所。妇儿、外侨、对台、保密、人防、档案、气象等工作取得新成绩。

　　社会保持和谐稳定。公安职业化、规范化、信息化建设全面推进，“一标三实”基础信息采集深入开展。基层民主和乡村治理机制建设稳步推进，第九届村（居）委会换届选举基本完成，井研成为全国一事一议规范管理县。乐山社区网开通运行，新设置社区工作站165个，社区服务体系更加完善。乐山市行政区划图正式出版。群众和信访工作得到加强，一批信访突出问题妥善解决。全面加强安全生产制度建设，深入开展隐患大排查大整治，安全生产形势持续稳定好转。退役士兵安置改革政策平稳实施。国防动员和后备力量建设质量有了新提高。民族宗教政策全面落实。

　　五、政府自身建设得到加强

　　一年来，我们修订完善市政府工作规则，积极转变政府职能，全面加强政府自身建设。

　　依法行政取得实效。“法治乐山”创建深入开展，第二届市政府法律顾问团组建成立，夹江成为全省依法行政先进县。坚持重大事项向市委报告制度，坚决执行市委的决定和工作部署。认真落实市人大及其常委会的决议、决定，积极采纳市政协的建议和调研成果，办理人大代表建议批评意见113件、满意和基本满意率99.1%；办理政协委员提案323件，满意和基本满意率100%。

　　作风建设深入推进。认真贯彻执行改进工作作风、密切联系群众各项规定，深入开展“实现伟大中国梦、建设美丽繁荣和谐四川”主题教育活动，集中开展“四反对四争当”作风建设活动，政风行风进一步改进。市本级“三公”经费支出在年初公开预算数基础上压缩10%，其他一般性支出压缩5%。市政府文件会议进一步精简。政务（便民）服务机构标准化、规范化、网络化建设扎实推进，电子政务服务功能逐步完善。政府绩效管理全面推行。加强政府系统廉政建设，营造了风清气正的发展环境。

　　各位代表：过去一年的成绩来之不易。这是省委、省政府和市委坚强领导的结果，是市人大、市政协和社会各界监督支持的结果，是全市广大干部群众共同奋斗的结果。我代表市人民政府，向全市各族人民，向驻乐部队指战员、武警消防官兵、政法干警，向各民主党派、工商联、无党派人士、各人民团体和各界朋友表示衷心的感谢，并致以崇高的敬意！

同时，我们也清醒地认识到，我市经济社会发展仍面临一些困难和问题，主要表现为：经济下行压力仍然较大，回升向好发展的基础还不牢固，部分经济发展预期目标未能实现；产业层次不高，结构调整任务艰巨，必须加快转型升级；城乡居民收入增长面临的制约因素增多，保障和改善民生任务依然艰巨；政府职能转变力度还需加大，公共服务效率需要进一步提高。对此，我们高度重视，正在采取有力措施认真加以解决。

2014年工作安排

　　2014年是在新的起点上全面深化改革的开局之年，是实施“十二五”规划承上启下的关键一年。市政府工作的总体要求是：紧紧抓住深入开展党的群众路线教育实践活动的契机，坚持科学发展、加快发展的工作指导思想，深入实施“三大发展战略”，紧扣“项目落地年”工作主题，以稳中求进为基调，改革创新为动力，加快转型为重点，项目投资为抓手，改善民生为根本，美丽发展为取向，全力稳增长、调结构、促改革、惠民生，进一步提高发展质量效益，增强发展活力后劲。主要目标是：地区生产总值增长9.5%，全社会固定资产投资增长14%，规模以上工业增加值增长10.5%，社会消费品零售总额增长13%，地方公共财政收入增长9.5%；城镇居民人均可支配收入增长10%，农民人均纯收入增长11%；全面完成省下达的各项目标任务。我们将重点抓好以下工作：

　　一、全面深化改革扩大开放

　　围绕增强发展动力活力，加强重点领域改革，全方位扩大开放合作，把改革创新贯穿于经济社会发展各个领域各个环节。

　　纵深推进“三大改革”。深化行政审批制度改革，动态调整精简行政审批事项，加强非行政许可审批项目和行政事业性收费清理，规范管理中介服务组织，推行并联审批、代办服务，优化审批流程，提高服务效率。深化农村产权制度改革，力争市中区等县（市、区）完成农村土地承包经营权确权登记颁证，支持井研开展农村土地流转收益保证贷款试点，加快建立农村产权交易平台，促进农村土地规范流转、规模经营。深化户籍管理制度改革，完善教育、住房、社保等配套政策，促进有能力在城镇稳定就业和生活的常住人口有序实现市民化。

　　拓展重点领域改革。紧紧围绕使市场在资源配置中起决定性作用和更好发挥政府作用，重点抓好经济体制改革，充分激发各类市场主体的发展活力。加快国有企业股权多元化进程，积极发展混合所有制经济。支持峨眉抓住列为全国首批旅游综合改革试点县（市）契机先行先试，支持马边申报国家旅游扶贫试验区，争取我市成为全国旅游综合改革试验区，着力推进旅游投融资体制、综合执法等专项改革，探索风景名胜区资源一体化管理和有偿使用制度。全面实施财政综合绩效管理，加强财源培植和建设，提高财政资金使用效益，开展市对县（市、区）收入划分体系改革，合理界定市、县两级事权与支出责任，切实防控政府性债务风险。扩大“营改增”试点。

　　务实推进开放合作。用好“中外知名企业四川行”和西博会等重大投资促进活动平台，积极组织开展各类专题招商活动。围绕产业链、供应链、价值链，着力抓好产业化项目专业招商、精准招商、园中园招商。力争引进到位市外内资500亿元，实际利用外资1亿美元。加强外向型经济园区和出口示范基地建设，完善乐山—东盟商务平台功能，力争实现外贸进出口总额12亿美元以上。支持有条件的企业“走出去”开拓国际市场。充分融入成德绵眉资乐雅遂同城化发展，主动对接长江经济带、丝绸之路经济带规划，积极扩大与沿海沿江沿边地区的经贸合作。

　　二、持续加大项目投资力度

　　围绕“项目落地年”工作主题，强化项目投资建设，用项目落地来承载发展的速度、质量和民生改善、社会进步。

　　全面推进“千亿项目”。加强项目落地开工、续建推进、建成达效全程服务，确保完成全社会固定资产投资897亿元。指导业主加快前期工作，促进意龙纺织工装产业孵化园、德诚商贸物流园等389个项目开工建设。积极协调解决项目推进中的困难和问题，推动科创生物医药产业园、都市农业示范带等215个续建项目快投快建，力争新万兴碳纤维复合材料、新长征药业园一期等273个项目竣工投用。密切跟踪招商签约项目，推动万达广场、乐山中科科技产业园等63个项目尽早落地建设。精准对接国家和省上投资导向，突出抓好技术创新、节能环保、民生保障等领域项目生成，动态充实项目储备库。

　　主攻重大项目建设。健全重大项目每月调度、季度协调、半年小结机制，完善看板管理、统筹协调、督查考核等制度，强化工作执行，有力推进重大项目建设，确保208个市列重大项目完成投资390亿元。完善重大项目领导挂联办法，对技术含量高、带动能力强、市场前景好的关键项目，实行“一名领导挂联、一套工作班子、一包到底服务”推进建设。加强项目包装推介，力争更多重大项目进入国家和省上“盘子”。

　　强化要素协调保障。大力破解用地瓶颈制约，积极开展城乡建设用地增减挂钩试点，探索发挥好农村集体经营性建设用地权能，盘活存量土地，实施差别化供地，集约节约用地。协调银企对接，力争全年新增贷款120亿元。鼓励优势企业抓住股票发行注册制改革、“新三板”扩容等政策机遇，多渠道开展股权融资，提高直接融资比重。做实政府性投融资平台，探索通过发行市政债、社会资本特许经营等方式，拓宽政府性工程建设融资渠道。提高煤电气等生产要素储运、调配能力，推动塘叶220千伏输变电工程等项目建成投运，力争枕头坝一级水电站并网发电。

　　三、加快工业转型升级步伐

　　围绕实施工业“双千双百”培育工程，强化工业主导地位，以科技创新支撑引领工业转型升级。

　　培育壮大主导产业。加快发展电子信息（物联网）、新能源、新材料、现代装备制造等新兴产业，抓好甲骨文实训基地暨软件产业园、中国华录·易华录西部产业基地等项目建设，力争新兴产业占全市规模以上工业企业主营业务收入的比重提高到35%。抓住国家支持光伏产业复苏契机，鼓励永祥多晶硅公司、乐电天威公司加快实施冷氢化技改，推进硅材料和光伏企业重组整合。统筹推进冶金建材、盐磷化工等传统产业技术改造和过剩产能化解，抓好华构住工新型城镇化示范项目、福华12万吨草甘膦扩能技改等项目建设，完成淘汰落后产能重点项目24个。

　　增强园区企业实力。实施“2244”园区培育计划，推动乐山高新区等4个省重点培育产业园区做强主业、提档升级，支持峨眉工业集中区加快调整发展，引导其他产业园区特色化、集约化发展，力争全市园区工业集中度提高到72%。支持市中区、五通桥、沙湾加快老工业基地调整改造，推动犍为嘉阳独立工矿区转型发展。围绕“2359”企业培育目标，实施大企业发展、中小微企业成长工程，力争新增主营业务收入超50亿元、20亿元企业各1家，净增规模以上企业20家。

　　大力推进科技创新。深入实施创新驱动发展战略，争创省技术创新工程示范市。强化企业创新主体地位，发挥龙头企业创新骨干作用，激活中小企业创新创造活力，新培育国家高新技术企业10家以上，省级创新型企业5家以上，技术创新投资增长15%。发挥乐山高新区、农业科技园区、硅材料产业化基地、五通桥可持续发展实验区、沙湾不锈钢产业园区等“国字号”平台示范引领作用，争创一批国家级和省级创新平台，打造区域创新集群。加强知识产权保护，加快推进科技成果转化，力争科技进步对经济增长的贡献率提高1.4个百分点。

　　四、提速发展现代服务业

　　围绕建设国际旅游目的地，大力发展以旅游为龙头的现代服务业，推动服务业发展提速、比重提高、水平提升。

　　力促旅游转型升级。按照“依托两核、建设两极、带动两线”的思路，完善景城一体化规划，推动发展千亿文旅产业，力争旅游综合收入增长20%。围绕食、住、行、游、购、娱等旅游要素，重点抓好一批旅游度假区、品牌酒店、实景演出、特色餐饮街区、旅游商品会展交易等项目，精心包装推出一批旅游产品和旅游精品线路，推动峨眉山国际旅游度假区、乐山大佛国际旅游度假区、中信国安峨眉国际旅游山城等项目建设，促成嘉定院子精品酒店、黑竹沟景区公路等项目竣工。创新旅游营销平台，加强旅游形象推广，承办第五届中国国际自驾游交易博览会，办好首届中国西部旅游博览会、第六届乐山大佛旅游文化节。实施旅游景区创建升级工程，力争仙芝竹尖生态园、嘉阳·桫椤湖创建为4A级旅游景区，新增省级以上水利风景区5个。推进旅游标准化建设，依法加强旅游市场监管，提高旅游服务质量。

　　优化提升服务业态。加快构建区域性商贸物流中心和现代服务业集聚区，抓好远成综合物流基地、九峰国际汽车博览城、西部不锈钢金属综合交易市场等项目建设，积极引进苏宁电器集团、保利集团等知名商贸品牌。大力发展电子商务，鼓励企业在知名电商平台开设“乐山造”产品专区，配套发展快递、连锁经营、第三方物流等业务。引导工业企业实施主辅分离，加快发展生产性服务业。进一步改善金融生态环境，加快发展金融产业，推进四川金融次中心建设。引导国有银行完善县、乡分支机构，支持市商业银行跨区域发展、加快做大做强。扶持发展健康、养老、文化等生活性服务业。

　　持续扩大城乡消费。规范收入分配秩序，引导发展普惠金融，努力满足消费信贷需求，多渠道提高城乡居民消费能力。加强市场供应保障，保持物价总水平基本稳定。整顿和规范市场秩序，切实改善居民消费环境。积极搭建促销平台，广泛开展农超对接、工业品下乡活动，繁荣消费市场。开展信息消费试点，支持电信运营商加强信息基础设施建设，丰富终端消费产品，全面提升社会信息消费水平。培育智能家电、新能源汽车等消费热点，合理发展住房、汽车等大宗消费。

　　五、毫不松懈抓好“三农”工作

　　围绕建设幸福美丽新村，认真落实强农惠农富农政策，全面促进农业增效、农民增收、农村发展。

　　大力发展现代农业。严格保护耕地，加强粮食生产，实施科学储粮，确保粮食总产量稳定在108万吨以上。推进青衣江乐山灌区续建配套与节水改造、都江堰井研灌区梅旺支渠等重点水利项目，实现金王寺水库工程下闸蓄水。实施山水田林路综合治理，确保新增有效灌面5万亩、高标准农田9万亩。加强现代农业（林业、畜牧业）重点县建设，推进特色优势产业规模化、标准化、品牌化。支持开展“峨眉山茶”全国知名品牌示范区创建活动，举办第四届“峨眉山茶”节。大力发展家庭农场、专业大户、农民合作社、产业化龙头企业等新型主体，鼓励农业生产经营方式创新。抓好农产品质量安全监管，支持开展“三品一标”农产品申报认证。

　　建设幸福美丽新村。修编完善新农村建设规划，分类推进彝家新寨、新农村综合体、新村聚居区建设，打造业兴、家富、人和、村美的幸福美丽新村。统筹抓好住房建设、基础设施、产业发展、公共服务和生态保护，加快改善农村生产生活条件，确保新（改）建县乡公路100公里、通村公路500公里，改造升级农村电网800公里以上，新建沼气池5200口，解决15万农村人口饮水安全问题。支持犍为、井研、夹江开展省级新农村建设示范县建设。

　　深入推进扶贫攻坚。全面落实扶贫开发纲要，整合资源、精准扶贫，力争4.15万贫困人口实现脱贫解困。深化小凉山—乌蒙山乐山片区和市列重点区域扶贫工作，大力实施“八大扶贫工程”，开展水、电、路、气、房、环境改善和科普“七到农家”行动，完成40个彝家新寨建设。抓好6个在建大中型水利水电工程移民安置工作，加强移民后期扶持项目建设。

六、扎实推进新型城镇化

　　围绕提高城镇化质量，坚持以人为核心，积极探索形态适宜、景城融合、产城互动、城乡统筹、集约高效的新型城镇化路子。

　　突出全域交通建设。全力以赴构建次枢纽，推动成绵乐铁路客专乐山段完成收尾工程，加快绕城高速、仁沐新高速仁寿至井研段、成贵铁路乐山段、成昆铁路新线成峨段建设，力争开工建设乐山机场、连乐铁路、乐汉高速峨眉至峨边段、仁沐新高速井研—沐川—马边段。加强地方重点交通项目建设，加快推进井沙、峨夹联网畅通工程，确保市公交客运总站、市长途客运总站、燕岗铁路货场扩建和省道306线峨眉至峨边段、省道103线沐川至马边段改造等项目建成投用。

　　精心构建“双百”城市。坚持用规划引领城市建设，提升规划编制水平、增强规划刚性，力争实现中心城区片区控规全覆盖。实施路网畅通工程，完成乐青路南段新建工程，开工建设乐沙城际生态大道等城市干道。实施新城再造工程，加快青江新区、岷江东岸、临港新城、苏稽片区、沙湾绿岛水城等基础设施及文旅、商业等配套功能建设，确保完成宝莲广场一期等项目建设。实施老城环境提升工程，完成老霄顶文化公园综合整治，推进竹公溪、滨江、滨河路等重点区域综合整治。加强人防工程建设，科学利用城市地下空间。提升城市园林绿化和风貌塑造水平，做好国家园林城市复查迎检工作。

　　提升城镇发展质量。优化城市生活、生产、生态布局和形态，推动大中小城市和小城镇协调发展。支持峨眉推进安川、城西新区项目，犍为开发方舟、牟家林新区，加快向中等城市迈进。支持抓好夹江漹江新区、井研城南新区、沐川幸福新区、马边红旗新区建设，启动一批试点示范镇建设，打造一批特色工业镇、旅游镇、商贸镇。加强市政基础设施和公共服务设施建设，提升城镇综合承载和吸纳就业能力，力争全市城镇化率提高1.5个百分点。办好新市民学校，帮助农业转移人口加快融入城市生活。创新城市管理体制机制，整合用好城市管理资源，统筹推进美丽城市、智慧城市、文明城市建设。支持有条件的地方实施乡改镇、镇改街。继续争取夹江撤县设区。

　　七、加强民生和社会建设

　　围绕保障和改善民生，不断完善民生制度设计，创新社会治理，让人民群众更多更好地共享改革发展成果。

　　实施重点民生事项。继续实施“十项民生工程”，办好一批民生实事。加强就业创业工作，积极帮助高校毕业生等重点群体实现就业，新增城镇就业3.8万人，促进6000人创业，力争创建为省级创业型城市。提高最低工资标准，健全农民工工资支付保障制度，落实企业退休人员养老金调待政策。健全城乡一体化社会保障制度，完善社保关系转移接续办法，统一农民工与城镇职工失业保险政策，落实省内异地就医同步结算制度。加强社保基金管理，保障基金安全、支付可持续。统筹推进百姓安居工程建设，确保开工公租房（含教师周转房）4000套、城镇危旧房和各类棚户区改造10586套，新（改）建农村住房15284户，基本完成城乡住房灾后重建任务。完善各类群体社会救助机制，适时提高城乡低保标准和农村五保供养水平。大力发展老龄事业，健全社会养老服务体系，试点建设信息化养老服务平台，推进市老年大学新建工程。建立重度残疾人护理补贴制度。全面实施新一轮妇女儿童发展纲要。建立关爱服务机制，给予农村留守儿童、留守妇女、留守老人更多照顾。

　　统筹发展社会事业。优化教育资源布局，加强中心城区中小学校扩容改造、农村义务教育薄弱学校改造、公办幼儿园建设，抓好犍为一中、夹江外国语实验学校迁建和乐山职业技术学院新校园等项目建设，确保峨边职教中心建成投用。推进公办中小学教师“县管校用”改革、考试招生制度改革，提高各级各类教育质量。加强医疗卫生服务能力建设，完成市人民医院综合楼、武警四川总队医院门诊大楼建设和市精神卫生中心迁建工程，推进疾病预防控制体系等级创建，加强彝区和基层医务人员培养。深化医药卫生体制改革，鼓励引导社会资本办医，试行全科医生特岗制度，探索建立社区医生和居民契约服务关系。启动实施“单独两孩”政策，把人口自然增长率控制在3.5‰以内。繁荣发展文体事业和文体产业，抓好市图书馆、市体育中心改扩建和乡村（社区）文化院坝等项目建设，实施中心城区有线数字电视整体转换，广泛开展群众性文体活动，积极组团参加省第十二届运动会、第八届残疾人运动会和第七届少数民族艺术节。完善邮政网底服务设施。完成第三次经济普查，配合开展第一次全国地理国情普查。加快档案数字化进程。做好外侨、对台、保密、防震减灾、地方志等工作。

　　加强生态文明建设。深入推进城乡环境综合治理，加快建设美丽乐山，争创全国生态文明先行示范区。加强生态保护，推进生态功能区、自然保护区、天然林保护区建设，广泛开展生态县（市、区）、生态园区创建活动。修复治理生态脆弱区，科学防御地质灾害。加强饮用水源地管理，支持五通桥、犍为开展省级节水型社会重点县（区）建设。抓好市第一污水处理厂扩建、市第三污水处理厂新建等减排项目，确保71处乡镇生活污水处理设施、20家规模化畜禽养殖场治理项目建成达效，力争开工市综合垃圾处理场。下大力气治理灰霾、小流域污染，狠抓工业排污、机动车尾气、重金属、农村面源等污染治理。

　　提高社会治理水平。扎实开展“六五”普法，深化法治乐山建设。加强城乡社区建设，提高社区信息化、网格化和社会化管理服务水平。开展未成年人社会保护试点。完善人民调解、行政调解、司法调解联动工作体系，及时化解社会矛盾纠纷。加强立体化社会治安防控体系建设，推进基层派出所建设和管理，依法惩治各类违法犯罪活动。建立最严格的覆盖全过程的食品药品监管制度，保障人民群众“舌尖上的安全”。强化安全生产监管，开展道路交通安全综合整治攻坚年活动，推进煤矿企业清理整顿和兼并重组，加快安全生产综合信息平台建设，深入排查治理各类安全隐患，切实把安全生产工作要求落实到基层一线、操作层面，促进安全生产形势持续稳定好转。完善防灾减灾救灾体系，提高突发事件预防预警和应急处置能力。依法维护网络信息安全，重视舆情引导，及时回应群众关切。认真贯彻党的民族宗教政策，开展峨边、马边彝族自治县成立30周年庆祝活动。加强国防教育、国防动员和后备力量建设，抓好军队转业干部和退役士兵安置工作，深入开展拥军优属和军民共建活动，争创第十届省级双拥模范城市。

　　以改革创新精神加强政府自身建设

　　新形势、新任务对政府工作提出了新的更高要求。我们将按照为民务实清廉的要求加强政府自身建设，全面正确履行政府职能，努力建设人民满意的法治政府和服务型政府。

　　坚持群众路线。按照“照镜子、正衣冠、洗洗澡、治治病”的总要求，深入开展党的群众路线教育实践活动。坚决反对形式主义、官僚主义、享乐主义和奢靡之风，完善改进作风常态化、长效化制度。从严控制政府发文和会议数量，倡导开短会、讲短话。完善直接联系和服务群众、民情观察员、网络对话、基层夜话等制度，深入开展走基层、挂帮包等活动，积极为群众排忧解难。加强和改进信访工作，统筹网上网下受理渠道，及时解决群众合理合法诉求。加快便民服务机构网络化、智能化建设，为群众提供优质、快捷服务。

　　加快职能转变。正确处理政府与市场、政府与社会的关系，最大限度减少政府对微观事务的管理，强化公共服务、市场监管、社会管理、环境保护等职责，提高政府管理科学化水平。完成新一轮市、县政府机构改革，严格控制机构编制。完善事业单位分类改革配套政策，推动公办事业单位与主管部门理顺关系和去行政化，完善事业单位法人治理结构。加大政府购买公共服务力度，开展政府购买事务性管理服务试点，发挥好志愿服务、公益慈善、行业协会等社会组织的积极作用。改进目标考核评价办法，统筹完善绩效管理、政务督查、行政问责等机制。

　　推进依法行政。加强市、县法治政府建设，推动政府公职人员学法制度化、常态化，提高运用法治思维和法治方式深化改革、推动发展、化解矛盾、维护稳定的能力。完善规范性文件、重大决策合法性审查机制，更好发挥政府法律顾问团作用，提高行政决策质量。深化行政执法体制改革，充实基层执法力量，完善行政执法程序，规范行政执法自由裁量权。认真贯彻执行市委决策部署，主动接受市人大及其常委会的法律监督和工作监督，自觉接受市政协的民主监督。广泛听取各民主党派、工商联、无党派人士和社会各界的意见。支持工会、共青团、妇联等人民团体开展工作。

　　深化廉政建设。推行政府及工作部门权力清单制度，依法公开权力运行流程。完善政务公开、办事公开、新闻发言人、政府网站建设等制度，开通市、县政务微博和微信公众账号，促进政民互动。严格控制“三公”经费支出，认真执行厉行节约反对浪费各项规定。深化制度建廉城市建设，加强工程建设、公共资源交易等重点领域和关键环节廉政监管，从源头上防治腐败。加强行政监察和审计监督，支持执纪执法机关依法查办案件。

　　各位代表：责任重于泰山，事业任重道远。让我们在省委、省政府和市委的坚强领导下，紧紧依靠全市人民，解放思想、锐意进取，爬坡实干、争创一流，奋力推进乐山追赶跨越、美丽发展，为谱写伟大“中国梦”精彩的乐山篇章而努力奋斗！
